# New edge of antibiotic development: antimicrobial peptides and corresponding resistance

**DOI:** 10.3389/fmicb.2014.00147

**Published:** 2014-04-08

**Authors:** Nadia S. Parachin, Octavio L. Franco

**Affiliations:** ^1^Grupo engenharia de Biocatalisadores, Departamento de Biologia Celular, Instituto de Ciências Biológicas, Universidade de BrasíliaBrasília, Brazil; ^2^Programa de Pós-Graduação em Ciências Genômicas e Biotecnologia, Centro de Análises Proteômicas e Bioquímicas, UCBBrasília, Brazil

**Keywords:** antimicrobial peptide, microbial resistance, antitumoral peptide, host defense peptide

In the last years severe efforts turned into extensive research on development of novel antimicrobial compounds. Among them, antimicrobial peptides, commonly isolated from several organisms, have been considered part of innate immune system and also as potential antimicrobial drugs. AMPs have variable amino acid composition and size (ranging from less than 5–100 amino acid residues), commonly showing cationic and amphipathic properties. Nowadays about 2300 AMPs have been reported in the Antimicrobial Peptide Database (AMP database). Such research endeavor resulted in more than 100 peptide-based drugs available in the market with approximately 500–600 candidates in pre-clinical test development (Craik et al., 2013). Therefore the current issue encloses several aspects when studying within antimicrobial peptides field.

Besides its antimicrobial activity, some AMPs also have antifungal activity, inmmunomodulatory and antitumural activities. For example defensins commonly found in plants, fungi, insect and mammalian cells are small cationic peptides of 45–54 amino acid residues with a conserved signature of cysteines, which can form three to four disulfide bridges. Those have been shown to have inmmunomodulatory activities that can be used as vaccine adjuvants or in the treatment of immune-depressed patients. Therefore, a better understanding of function and mechanism of action of such molecules is a great promise in anti-infective and immunomodulatory therapeutics (Silva et al., 2014).

Defensins isolated from plants also have been considered as a biotechnological tool to improve crop production as recently reviewed (Lacerda et al., 2014). Among their advantages low effective fungicide concentrations and decreased environmental impact have been focused when compared to most common chemicals utilized for fungi control. Therefore many studies have focused in construction of genetic modified plants that over produce such molecules in order to reduce crop losses by fungi infection. Nevertheless it is important to emphasize that although there are many studies occurring at bench scale, indicating that genetic modified plants over producing defensins should appear in the market within the next years (Lacerda et al., not published).

It is also know that antimicrobial peptides have other potential applications since some of them have also antitumoral and imunomodulator activities. Also know as anticancer peptides (ACP) and host defense peptides (HDP) they also represent important candidates as novel drugs and have been carefully addressed in the current issue. For instance, cationic peptides have been considered as anticancer agents for presenting numerous advantages over chemical agents such as higher specific cytotoxicity to tumor cells, lower side effects and easier absorption as recently listed (Mulder et al., 2013). Diverse studies have shown that cancer cells are more anionic than normal cells. Due this property, cationic AMPs seems to bind it faster and selectively resulting in cell death. A better understanding of ACP mechanism of action may result in novel pharmacies with optimized anticancer activity (Gaspar et al., 2013).

Despite the great number of peptides available in databases, isolation of new molecules using classic purification techniques is crucial to identify novel molecules with diverse activities. Indeed a proteinase inhibitor isolated from *J. curcas* seed cake, named JcTI-I, was shown to have a potent activity against the human pathogenic bacteria *S. aureus* and *S. enterica*. Moreover it did present other relevant pharmacologically characteristics such as absence of hemolytic activity against human erythrocytes concomitant to pH and high salt concentration resistance (Costa et al., 2014). Moreover, microorganism have also been focused in this issue were the isolation and characterization of lipopeptide from *Bacillus* sp. were performed. This lipopetide shows strong fungicide activity (specially when this compound was self-assembled) (Roy et al., 2013).

Lately not only nature has become a source of AMPs. Besides isolation of natural organisms, antimicrobial peptides might be improved or created using computational tools. This opens even more this so amazing field by creating infinite novel and remarkable possibilities. Recently a study screened the distribution of known motifs in prokaryotic extracellular and virulence proteins across a range of bacterial species in order to identify novel motifs in virulence proteins (Ruhanen et al., 2014). Such methodologies are able to generate thousands of novel molecules that require high-throughput *in vitro* and *in vivo* validation. In this sense it is also necessary to develop rapid assays that can be performed concomitant to million candidates. A recent method enabled the selection of AMPs directly on peptide microarrays allowing identification of AMPs that bind and are bactericide among those that bind but do not kill bacteria (Wimley, 2010). In another example, an affinity support might be applied for the isolation of AMPs that interact with lipopolysaccharides, the major cause of septic shock (Lopez-Abarrategui et al., 2013). Thus advances in peptide array discovery assays could provide a system to develop pathogen-specific antibiotics resulting in the discovery of target antibiotics (Diehnelt, 2013).

In alignment with *in silico* models, several systemic strategies such as transcriptome and proteome have been utilized for understanding peptide interaction with its target, which may allow improvement or design new molecule properties (Tavares et al., 2013). These novel approaches could lead us to understand several compounds at the same time which may result in a rapid increased in AMP peptides available in the market for treating several diseases. Finally it is fundamental to understand mechanism of action of AMPs in order to improve its activity and predict possible resistance mechanisms. Although many AMP alters membrane permeability there are other action mechanisms such as synthesis inhibition of cell wall, protein synthesis or nucleic acids as reviewed in this issue (Guilhelmelli et al., 2013). Moreover it is also essential to clear elucidate host-pathogen relationship. A recent study hypothesized that Helmint defense peptides played a critical role in parasite interaction with its host (Robinson et al., 2013). This may be applied as immunomodulators agents since those molecules are able to interact with the host without cytotoxic or cytolytic effects.

Overall the current issue highlights the relevance of such research topic with perspectives to develop entirely new molecules with vast application within health and agricultural field with c higher affinity for its target with concomitant reduction of side effects.

## Conflict of interest statement

The authors declare that the research was conducted in the absence of any commercial or financial relationships that could be construed as a potential conflict of interest.
